# *Citrobacter amalonaticus* Phytase on the Cell Surface of *Pichia pastoris* Exhibits High pH Stability as a Promising Potential Feed Supplement

**DOI:** 10.1371/journal.pone.0114728

**Published:** 2014-12-09

**Authors:** Cheng Li, Ying Lin, Yuanyuan Huang, Xiaoxiao Liu, Shuli Liang

**Affiliations:** Guangdong Key Laboratory of Fermentation and Enzyme Engineering, School of Bioscience and Bioengineering, South China University of Technology, Guangzhou, 510006, P. R. China; CNRS, France

## Abstract

Phytase expressed and anchored on the cell surface of *Pichia pastoris* avoids the expensive and time-consuming steps of protein purification and separation. Furthermore, yeast cells with anchored phytase can be used as a whole-cell biocatalyst. In this study, the phytase gene of *Citrobacter amalonaticus* was fused with the *Pichia pastoris* glycosylphosphatidylinositol (GPI)-anchored glycoprotein homologue *GCW61*. Phytase exposed on the cell surface exhibits a high activity of 6413.5 U/g, with an optimal temperature of 60°C. In contrast to secreted phytase, which has an optimal pH of 5.0, phytase presented on the cell surface is characterized by an optimal pH of 3.0. Moreover, our data demonstrate that phytase anchored on the cell surface exhibits higher pH stability than its secreted counterpart. Interestingly, our *in vitro* digestion experiments demonstrate that phytase attached to the cell surface is a more efficient enzyme than secreted phytase.

## Introduction

Phosphorus is stored in many cereals and oilseeds (approximately 1–3%) as phytic acid [1]. Although phytic acid plays an important role in many physiological activities, including the regulation of inorganic phosphate, the complexation of multivalent cations, competition for ATP and energy storage, this molecule is considered to be an anti-nutrient due to its strong ability to interact with proteins and starch or to chelate divalent minerals, such as iron and zinc [2]. In environments with pH values that range from acidic to neutral (i.e., pH values similar to those of the gastrointestinal tract), the chelation of metal ions is a common phenomenon [3]. Because chelation involves many minerals that are essential for the activity of intracellular and extracellular enzymes, serious metabolic disorders might occur as a consequence of excessive chelation [4].

Phytic acid can be degraded by phytase (*myo*-inositol hexakisphosphate 3- or 6-phosphohydrolases; EC 3.1.3.8 or EC 3.1.3.26). This enzymatic degradation can be facilitated by the addition of phytase isolated from natural sources or by supplementation with recombinant enzymes produced in various hosts, such as fungi and bacteria. Undigested phytic acid is excreted by monogastric animals due to the absence of a sufficient level of phytase in their digestive tracts [3]. This phenomenon may lead to eutrophication. Numerous countries have used phytase as a feed additive. The FDA has also approved a GRAS (Generally Recognized As Safe) petition concerning the use of phytase in food [5]. The application of phytase in animal feed lowers phytic acid levels and may also reduce feeding costs by reducing the requirement for supplementation with inorganic phosphorus [6] and reduce pollution caused by fecal excretion of phosphorus [7]. The most effective phytases are produced by microorganisms that reside inside animal tracts. To date, the phytases from several microorganisms and plants have been purified and characterized [8]. Among these phytases, phytase from *Citrobacter amalonaticus* CGMCC 1696 exhibited high enzymatic activity in *Pichia pastoris* [9].

Unfortunately, phytases from the majority of microorganisms are sensitive to pH and heat, which limits their application in the feed industry [10]. Moreover, many studied phytases express low specific activity in the desired environment; together with the low stability of these phytases, this characteristic results in high manufacturing costs [11]–[13]. Modifications of phytases that will enhance thermal or pH stability and increase specific activity will facilitate the application of these enzymes in animal feed and food processing [14], [15]. Cell surface technology can also circumvent some of these problems [16]. In cell surface technology, the target protein is displayed on the surface of either a phage or a cell in the form of a fusion protein. The production process is simplified when using cell surface technology, as no purification of the target protein is necessary [17]. Moreover, it was reported that proteins produced using cell surface technology may exhibit enhanced thermal stability [18], [19]. Phytases from *Aspergillus niger* BCC18081 [20] and *A. niger* NRRL3135 [21] have been expressed on the cell surface of *P. pastoris* and *Saccharomyces cerevisiae*, respectively. Although these yeast strains were successfully applied as whole-cell supplements, the phytase activity of these strains was lower than that of the secreted phytase. Moreover, phytases displayed on the surface of yeast cells were characterized by low pH stability.

Many anchored glycoproteins, such as agglutinins [22], Flo1p [23] and Cwp1p [24], are embedded in the lipid bilayer, with the bulk of their structure located on the yeast cell wall. Prior to the identification of glycosylphosphatidylinositol (GPI)-anchored glycoproteins, heterologous anchored glycoproteins were used for cell surface technology in *P. pastoris* [25]. GPI-anchored glycoproteins form a group of structural components, surface receptors and hydrolytic enzymes that play an important role in the formation of flocs, mats and biofilms [26], [27]. GPI-anchored proteins contain an N-terminal signal peptide that is required for ER targeting, and the C-terminus of these proteins is modified by the addition of a GPI anchor at a residue referred to as the omega (ω)-site [28]. In their signal sequences, GPI-anchored proteins usually contain a sequence that is enriched in Ser/Thr and provides sites for O-glycosylation. Li Zhang et al [25] reported that the hypothetical protein Gcw61p was the most abundant protein displayed on the cell surface among the identification GPI-anchored glycoproteins.

In this work, we expressed the phytase gene *Phy* from *C. amalonaticus* as a fusion protein on the surface of *P. pastoris* cells. We characterized the biochemical properties of the expressed cell surface phytase. Interestingly, our data demonstrate that the phytase expressed on the surface of *P. pastoris* cells displays high enzymatic activity and stability. Furthermore, we successfully used yeast cells expressing phytase on their surface as a whole-cell feed additive.

## Materials and Methods

### Strains, media and growth conditions

*Escherichia coli* TOP10F′ (Invitrogen, Carlsbad, CA, USA) cells were used for DNA manipulations; these cells were cultivated in low-salt LB medium. Bacterial plasmid selection and maintenance was performed using 25 mg/L of zeocin (Invitrogen, Carlsbad, CA, USA). The *P. pastoris* strain GS115 (Invitrogen, Carlsbad, CA, USA) was used as a host cell, and this strain was cultivated in YPD medium (1% yeast extract, 2% peptone and 2% glucose). Transformants of *P. pastoris* were selected on YPDSZ agar plates (1% yeast extract, 2% peptone, 2% glucose, 18.2% sorbitol, 2% agar and 100 mg/L of zeocin). The DNA segments encoding phytase with a Flag-Tag and the anchored glycoprotein gene *GCW61* were ligated into pPICZαA (Invitrogen, Carlsbad, CA, USA).

### Construction of vectors

Phytase (*Phy*) from *C. amalonaticus* CGMCC 1696 (GenBank accession number ABI98040.1) was generated using the primers PhyF (S1 Table, with an *EcoR* I restriction site and a Flag-Tag at the 5′ end) and PhyR (S1 Table, with a *Kpn* I restriction site) and cloned into the pPICZαA (ZαA) expression vector, resulting in plasmid pPICZαA-phy (Phy). The *GCW61* (REFSEQ accession number XM_002494287.1) gene was amplified from the *P. pastoris* genome using the primer pair GCW61F/GCW61R (S1 Table) and ligated into the *Kpn* I/*Not* I sites in the plasmid pPICZαA-phy to create plasmid pPICZαA-phy-GCW61 (Phy-GCW61). All plasmids were transformed into chemically competent *E. coli* TOP10F' cells and confirmed using restriction enzyme digestion and DNA sequencing.

### Yeast transformation

Plasmids were linearized using *Sac* I (Takara, Japan), which cuts in the *AOX*1 promoter, and transformed into *P. pastoris* GS115 competent cells via electroporation using a Gene Pulser apparatus (Bio-Rad, USA) with the following parameters: 1500 V, 25 *µ*F, and 200 Ω in a 0.2 cm cuvette. Electroporation was performed according to the manufacturer's instructions (Invitrogen). The transformed cells were selected on YPDSZ agar plates, which had been incubated at 30°C for 2–3 days.

### Cultivation of *P. pastoris* and expression of phytase

*P. pastoris* transformants were inoculated into 5 mL of BMGY medium (1% yeast extract, 2% peptone, 1.34% YNB, 0.00004% biotin, 100 mM potassium phosphate (pH 6.0) and 1% glycerol) in a 50 mL Erlenmeyer flask. The cells were precultivated overnight at 30°C and 250 rpm. Next, the main cultures were inoculated from precultures to obtain an initial optical density of 0.5. The cells were grown in 20 mL of BMMY medium (1% yeast extract, 2% peptone, 1.34% YNB, 0.00004% biotin, 100 mM potassium phosphate (pH 6.0) and 1% methanol) in a 250 mL Erlenmeyer flask in a shaking incubator at 30°C and 250 rpm. Fresh methanol was added to obtain a final concentration of 1% (v/v) every 24 h. OD_600_ and phytase activity were monitored throughout a 5-days incubation.

### Fluorescence microscopy and flow cytometry analyses

*P. pastoris* cells were induced for 96 h in BMMY, and the cells were subsequently harvested. The immunolabeling analyses were performed according to the method described by Kobori et al [29]. The harvested cells were washed twice in ice-cold water and resuspended in ice-cold phosphate-buffered saline (PBS, pH 7.4), with 10 mg/mL of bovine serum albumin to block the cell surface. Next, a monoclonal antibody against Flag-Tag (Agilent, USA) was used as the primary antibody. The cell suspension was incubated with the primary antibody at a dilution of 1∶200 in a total volume of 200 µL at room temperature for 2 h. Next, the cells were washed twice with PBS and exposed to the secondary Alexa Fluor 488 goat anti-mouse IgG (H+L) antibody (Invitrogen, USA) at a final concentration of 10 ng/µL for 1 h at room temperature. The cells were washed three times with PBS and analyzed using fluorescence microscopy (BX51, Olympus, Japan). In addition, the cell suspension was examined using flow cytometry (Beckman-Coulter, Fullerton, CA, USA). A total of 10,000 cells from each sample were analyzed. The data were processed using EXP032 software (Beckman-Coulter). GS115/ZαA were also processed in the same manner to serve as negative controls.

### Yeast cell wall isolation

The cell walls of yeast cells were isolated according to the method described by Schreuder [30], with modifications. The yeast cells were harvested by centrifugation after a 96 h induction and washed three times with ice-cold isolation buffer (10 mM Tris-HCl, pH 8.0, containing 1 mM phenylmethanesulfonyl). Next, the cells were mixed with isolation buffer and glass beads with a 0.5 mm diameter at a ratio of 1∶2∶1 (wet wt/vol/wt) in a microcentrifuge tube. The cells were disrupted by vigorous shaking using a vortex mixer. Shaking was performed at maximum speed for 30 s, with eight repeats separated by 1 min intervals of incubation on ice. The fraction containing the cell wall was isolated by centrifugation for 5 min at 6000×*g* and 4°C. The cell wall fraction was washed three times with 1 mM PMSF and resuspended in 200 µL of reaction buffer (100 mM sodium acetate, pH 5.0, and 1 mM PMSF). Next, 10 mU of laminarinase (Sigma, USA) per 100 mg of cell wall fraction (wet weight) were added. The mixture was incubated at 37°C for 2 h. Subsequently, an additional 10 mU of laminarinase was added to the mixture and the incubation was continued for another 2 h. Prior to determining the phytase activity, the supernatant was collected by centrifugation for 5 min at 10000×*g* and 4°C.

### Phytase Enzymatic Activity

Phytase activity was analyzed according to the method described by Žyla [31], with modifications. Fifty microliters of culture were centrifuged for 1 min at 10,000 g and room temperature. The cells were washed three times with 100 mM sodium acetate buffer (pH 5.5) to remove traces of phosphorus. A 1 mL volume of 100 mM sodium acetate buffer (pH 5.5) was used to suspend the cells, and the cellular suspension was preheated at 37°C for 5 min. Next, 2 mL of 5.0 mM sodium phytate (100 mM sodium acetate, pH 5.5) was added and the mixture was incubated at 37°C for 30 min. Next, 2 mL of coloration solution [24% nitric acid, 100 g/L of ammonium molybdate, and 2.35 g/L of ammonium vanadate, 2∶1∶1 (vol/vol/vol)] was added and the reaction was incubated for 10 min. The absorbance of the mixture was measured at 415 nm. One unit of activity (U) was defined as the amount of enzyme that hydrolyzed 5.0 mM sodium phytate per min to generate 1 *µ*mol inorganic phosphorus at 37°C. GS115/ZαA were also processed in the same manner to serve as background samples.

### Optimal pH and Temperature of cell surface phytase

To determine the effect of pH on the cell surface-exposed phytase, different pH values (100 mM glycine-HCl buffer, pH 1.6, 2.0, 2.5, 3.0, 3.5 and 4.0; 100 mM sodium acetate buffer, pH 4.5, 5.0, 5.5 and 6.0; and 100 mM Tris-HCl buffer, pH 7.0 and 8.0) were used. The optimal temperature was determined to be in the range of 30–90°C in 100 mM sodium acetate buffer, pH 5.5.

### pH and Thermal Stability of the Cell surface Phytase

To determine pH stability, the enzymes were preincubated at 25°C for 6 h in buffers with pH values ranging from 1.6 to 6.0 (100 mM glycine-HCl buffer, pH 1.6–4.0; 100 mM sodium acetate buffer, pH 4.5–6.0). Enzymatic activity was measured at 37°C in 100 mM sodium acetate buffer, pH 5.5.

To determine thermal stability, the enzymes were preincubated at 60–90°C for 5–120 min in 100 mM sodium acetate buffer, pH 5.5. Enzymatic activity was measured at 37°C in 100 mM sodium acetate buffer, pH 5.5.

### Effect of Metal Ions

The effect of metal ions was determined by measuring the cell surface phytase activity in 100 mM acetate buffer, pH 5.5, at 37°C with 5 or 10 nM Ba^2+^, Ca^2+^, Co^2+^, Cu^2+^, Fe^2+^, Fe^3+^, K^+^, Li^+^, Mg^2+^, Mn^2+^, Na^+^, Ni^2+^ or Zn^2+^.

### *In vitro* digestibility test

An *in vitro* digestibility test was performed to investigate the ability of recombinant phytase to digest phytic acid in corn-based animal feed in the presence of pepsin and pancreatin. According to the method of Zyla [32], the *in vitro* digestion tests simulated the pelleting process of animal feed and the temperature and pH of digestive conditions in the crop. A total of 1.5 mL of diluted enzyme solution (i.e., harvested cells or enzymes diluted with 100 mM acetate buffer, pH 5.9) was mixed with 1 g of corn-based feed and incubated at 40°C for 30 min to simulate digestion in the crop. Next, 0.5 mL of pepsin (Sigma, USA) solution (0.1 mol/L HCl containing 0.016 g/L of pepsin) was added in combination with 1.5 M HCl to regulate the pH to 2.9. Next, the mixture was incubated at 40°C for 45 min. Subsequently, 0.45–0.5 mL of 1 M NaHCO_3_ containing 3.7 mg/mL of pancreatin (Sigma, USA) was added to achieve a pH of 6.1. The mixture was incubated at 40°C for 2 h. The digested slurry was then filtered through filter paper (Whatman^R^ No. 1), and the amount of released phosphorus was determined. To simulate the pelleting process of animal feed, the enzyme/corn-feed mixture was preincubated at 80°C for 3 min or 90°C for 5 min. Next, *in vitro* digestibility tests were performed using either cells or secreted phytase at 4 U of phytase activity g^−1^ of feed. The samples were then compared to reference samples that were lacking cells or to secreted phytase. GS115/ZαA were also processed in the same manner to serve as background measurements.

## Results and Discussion

### Construction of a *P. pastoris* Surface Display System

The phytase gene *phy* was displayed on the cell surface of *P. pastoris* as a fusion with the *GCW61* gene product. To obtain an expression plasmid, an approximately 1300 bp fragment of *phy* was cloned into the pPICZαA (ZαA) expression vector using the *EcoR* I and *Kpn* I restriction sites (S1 Figure). Next, an approximately 160 bp fragment of the anchored glycoprotein gene *GCW61* was ligated with the pPICZαA-phy vector using the *Kpn* I and *Not* I restriction sites (S2 Figure). Two expression vectors, which were named Phy and Phy-GCW61, were constructed, and the basal vector was named ZαA. The obtained plasmids (i.e., Phy-GCW61, Phy and ZαA) were transformed into *P. pastoris* GS115. Correct integration of the constructs into the *P. pastoris* genome was verified using colony PCR with the 5′AOX and PhyR primer pair and the 5′AOX and GCW61R primer pair (S3 Figure).

**Figure 1:**
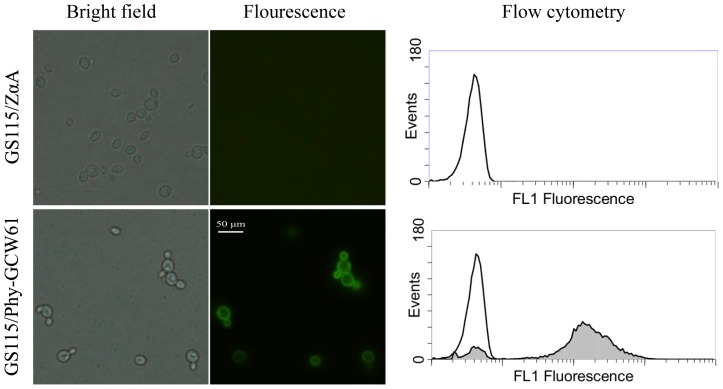
Fluorescence microscopy and flow cytometry analyses of yeast cells.

After 96 h of induction with methanol, the cells were harvested. The presence of phytase on the cell surface of *P. pastoris* was verified using indirect immunofluorescence and flow cytometry (Fig. 1). As a result of immunolabeling with an anti-FLAG antibody, the green fluorescent signal was observed in nearly all cells harboring plasmid Phy-GCW61; in contrast, little fluorescence was emitted by the control strain GS115/ZαA. The results from immunofluorescence experiments were confirmed via flow cytometry analyses (Fig. 1).

### Expression of phytase on the cell surface of *P. pastoris*

**Figure 2:**
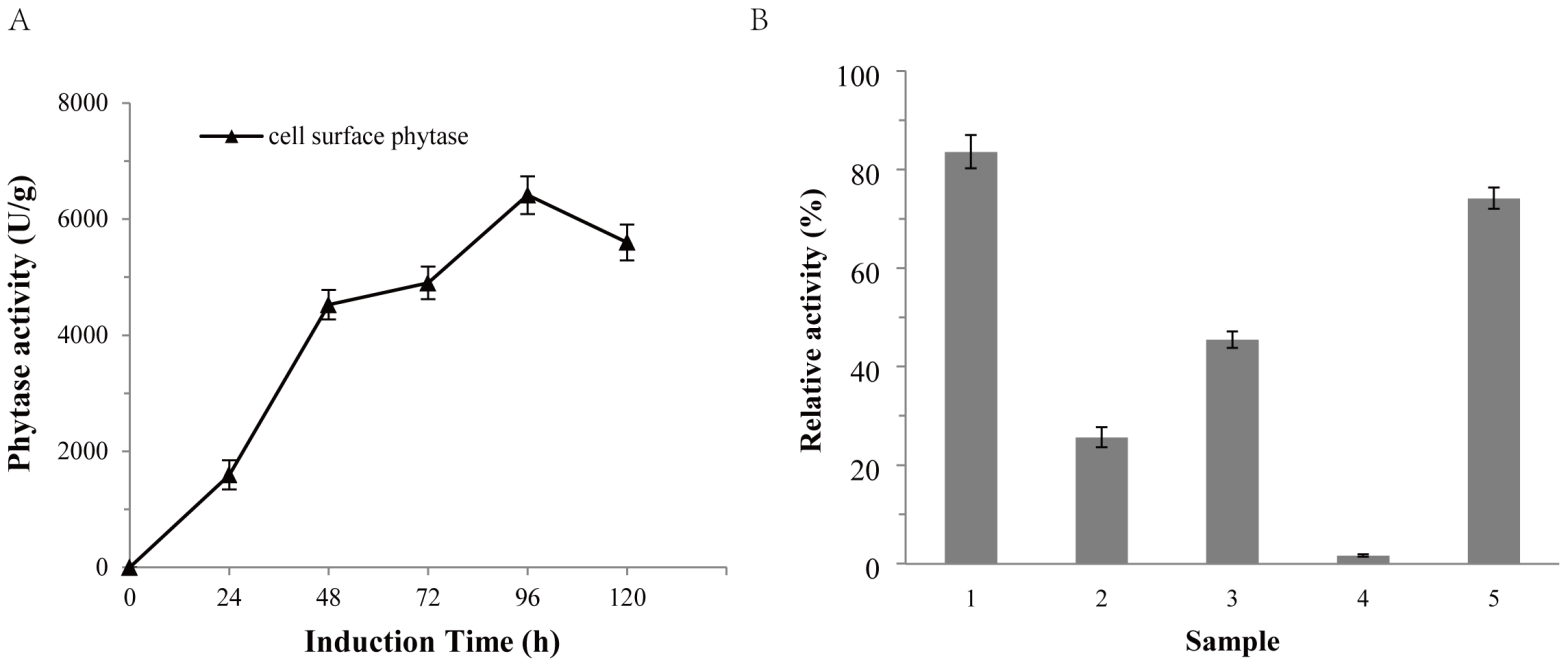
Phytase activity after induction with methanol and after treatment with laminarinase. A: Time dependence of the activity of cell surface phytase after induction with methanol. B: Cell surface phytase activity after laminarinase treatment. Column 1 represents cell wall fractions without treatment with laminarinase. Columns 2 and 4 represent cell wall fractions after laminarinase treatment, and column 3 and 5 represent supernatant fractions after laminarinase treatment. Columns 2 and 3 show phytase activities after treatment with 5 mU of laminarinase, while columns 4 and 5 represent the remaining activities after treatment with 50 mU of laminarinase. All activities were compared to the activity of the whole cell surface phytase, with GS115/ZαA as a background measurement.

The activity of cell surface phytase (6413.5 U/g cell dry weight) was observed after 96 h of induction with methanol (Fig. 2A). The obtained phytase activity was much higher than that of the previously studied phytase from *A. niger* BCC18081 on the cell surface of *P. pastoris* (approximately 300 U/g cell dry weight) [20]. The higher activity of *C. amalonaticus* phytase on the cell surface than that of *A. niger* BCC18081 may relate with its specific activity and the anchored protein. The display efficiency of heterologous proteins on cell surface could be affected by the characteristics of host cell, passenger protein, anchored protein and fusion method [33]. Although the phytase of *A. niger* BCC18081 has been expressed in *P. pastoris* [20], [34], its specific activity remains unclear. Nevertheless, the high specific activity (3548 U/mg [9]) of *C. amalonaticus* phytase is an important cause for the high activity on the cell surface of *P. pastoris.* Furthermore, the ability of different anchored proteins to display the same passenger protein on the cell surface varies [35]. It's worth noting that the display of *A. niger* BCC18081 phytase on *P. pastoris* cell surface was performed with *S. cerevisiae* α-agglutinin [20]. The anchored protein Gcw61p, used in the present study, displayed much more *Candida antarctica* lipase B (CALB) on the cell surface of *P. pastoris* than other 12 homologous GPI-anchored proteins [25] and α-agglutinin, a heterologous anchored protein [36]. Actually, we previously used the GPI-anchored glycoprotein Gcw51p [25] to display *C. amalonaticus* phytase and obtained only 30% of the Gcw61p displayed phytase activity on *P. pastoris* cell surface (Data not shown). Taken together, the high level of *C. amalonaticus* phytase on the cell surface may result from its high specific activity and the strong display efficiency of the anchored protein Gcw61p.

To ensure that phytase was anchored on the cell wall via GPI-anchored glycoproteins, laminarinase probing was performed. Laminarinase can hydrolyze β-1, 3 glucan bonds, including bonds that anchor glycoproteins to the cell wall. After treatment with laminarinase, the phytase activity in cell wall fractions decreased; this activity was then detected in the supernatant (Fig. 2B). With increasing amounts of laminarinase, the phytase activity further decreased in the cell wall fractions and increased in the supernatant. These data demonstrate that the cleavage of β-1, 3 glucan bonds disrupts the association of phytase with the cell wall and confirms that phytase is anchored to the surface of yeast cells via glycoproteins.

### Effects of Temperature and pH

**Figure 3:**
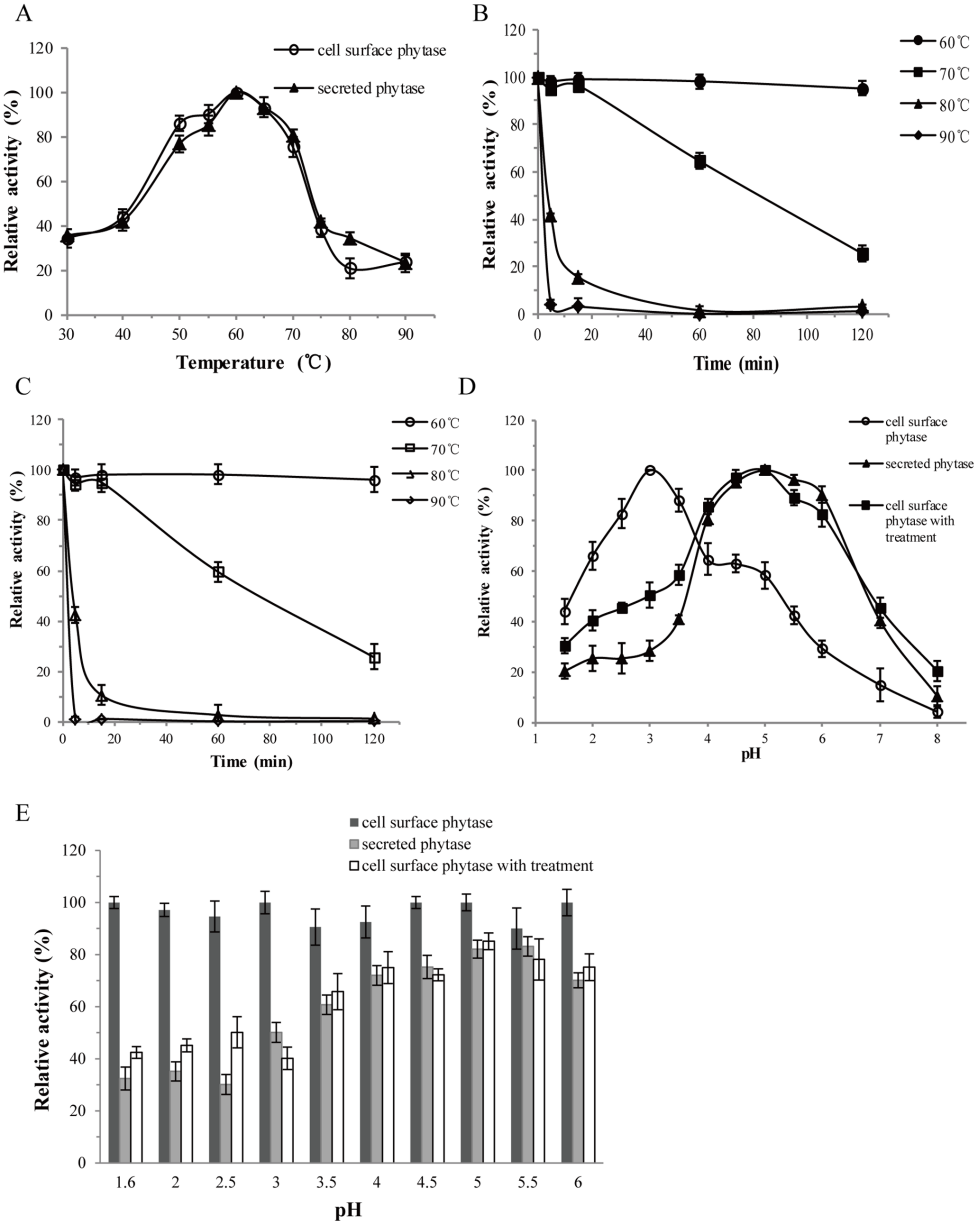
Effect of temperature and pH on the activity of the cell surface and secreted phytases. A: Activity of cell surface and secreted phytases at different temperatures. B: Thermostability of cell surface phytase. C: Thermostability of secreted phytase. D: Activity of cell surface phytase, secreted phytase and the supernatant fractions of cell surface phytase treated with 50 mU of laminarinase at different pH values. E: The pH stability of cell surface phytase, secreted phytase and the supernatant fractions of cell surface phytase treated with 50 mU of laminarinase.

The effect of temperature on the activity of the cell surface is shown in Fig. 3A. The optimal temperature for cell surface phytase and secreted phytase is 60°C, which is similar to data reported for phytases from *Candida melibiosica* 2491 [37] and *Pichia anomala* [38]. Phytase expressed on the surface of yeast cells and its secreted counterparts were stable when incubated at 60–70°C. Both of these proteins had high thermal stability, as>90% of the activity remained after incubation at 60°C for 2 h (Figs. 3B and 3C). More than 60% of the activity remained after 1 h of incubation at 70°C (Figs. 3B and 3C). Phytase from *A. niger* BCC18313 expressed on the surface of *P. pastoris* cells retained approximately 40% of the observed basal activity after incubation at 60°C for 1 h [20], suggesting that phytase from *C. amalonaticus* expressed on the surface of the same yeast host is characterized by higher thermal stability than its fungal homologue.

The optimal pH of the cell surface phytase was approximately 3.0 (Fig. 3D), while the optimal pH of secreted phytase and phytase released from the cell surface (i.e., treated with 50 mU of laminarinase) was 5.0. The optimum temperature and pH of the secreted *C. amalonaticus* phytase in *P. pastoris* were different with that described by Luo et al [9]. *Sna*B I (5′-TACGTA-3′, encoding a tyrosine and valine) and *Eco*R I (5′-GAATTC-3′, encoding a glutamic acid and phenylalanine) were added at the upstream of *C. amalonaticus* phytase coding sequence in the research described by Luo et al [9] and the present study, respectively. Besides, a Flag-Tag was added at the upstream of *C. amalonaticus* phytase coding sequence in the present study. The different restriction enzyme cutting sites and addition of the Flag-Tag resulted to different N-terminal residues of the secreted phytase in *P. pastoris*. Generally, the N-terminal residues of the protein could affect its enzymatic properties. The heat tolerance of *Piromyces rhizinflata* 2301 cellulase was significantly increased by adding a 28-residue non-catalytic domain to the N-terminus [39]. The turkey pancreatic lipases expressed in *P. pastoris* with or without N-terminal His-tag (consisting of six consecutive histidine residues) showed different stereospecificity at high surface pressures [40]. Besides, the N-terminal mutations of R24 and E17 of N-Acetylglutamate kinase (NAGK) in *Pseudomonas aeruginosa* caused diverse affinity for arginine [41]. Similarly, the different optimum temperature and pH of phytase between the present study and Luo et al [9] may relate to the different residues of N-terminal.

Interestingly, cell surface expression improves the stability of phytase in varying pH conditions. The cell surface phytase was stable at a broad range of pH values from 1.6 to 6.0 (Fig. 3E). In this pH range, cell surface phytase retained 90% of its activity while secreted phytase displayed only 70% of the observed basal activity in the pH range from 4.0 to 6.0. Using the GPI-anchor system, the lipase from *Rhizopus oryzae* with a pro sequence (ProROL) [18] and CALB [19] that were anchored on the cell surface of *S. cerevisiae* and *P. pastoris* exhibited higher thermal stability than the corresponding free enzymes. And we previously used the GPI-anchored glycoprotein Gcw51p [25] fused with the gene *Phy* and demonstrated increased pH stability similar to that of Gcw61p (data not shown). The mechanism and details of these phenomena remain to be investigated. Although site-directed mutagenesis can improve activity at low pH [14], cell surface technology can avoid protein purification and achieve whole-cell catalysis [42]. The observed stability of cell surface expressed phytase in extreme pH values may help to improve the activity of phytase in the digestive system.

### Effect of Metal ions

Both cell surface phytase and secreted phytase were strongly inhibited by 5 nM or 10 nM Cu^2+^ (18%–30%) and moderately inhibited by 5 nM or 10 nM Co^2+^ (60%–80%) (Table 1) but the secreted phytase was activated by high concentrations of Co^2+^ (1000 nM) [9]. Cu^2+^ might bind to the enzyme and form insoluble metal-phytase complexes. Interestingly, phytases were activated by 5 nM Ba^2+^, Ca^2+^, K^+^, Mg^2+^, and Na^+^. This observation is similar to previous reports of r-PhyA170 from *A. niger* BCC18313 in *P. pastoris* [20]. The presence of 5 nM Mn^2+^, Li^+^ and Fe^3+^ and 10 nM Ca^2+^, Fe^3+^, Na^+^, Zn^2+^, Ni^2+^ and Mn^2+^ moderately inhibited cell surface phytase. In 5 nM K^+^, the activity of cell surface phytase increased by nearly 50%. The observed increase in activity was less pronounced for secreted phytase (only 5%). When in 10 nM Co^2+^ and Li^+^, the activity of the secreted phytase decreased more than that of the cell surface phytase; in contrast, the opposite held true in 10 nM Ca^2+^, Fe^3+^, Mn^2+^, Ni^2+^ and Zn^2+^.

**Table 1:** Effect of metal ions on cell surface and secreted phytases.

Metal ions^a^	Relative activity (%)			
	5 nM		10 nM	
	secreted phytase	cell surface phytase	secreted phytase	cell surface phytase
none*^b^*	100	100	100	100
Ba^2+^	103.25±2.06*^c^*	108.79±1.32 *^c^*	97.63±2.20 *^c^*	114.57±2.78 *^c^*
Ca^2+^	108.51±2.35 *^c^*	105.04±0.62 *^c^*	85.31±2.02 *^c^*	77.28±1.04 *^c^*
Co^2+^	72.19±1.04 *^c^*	78.02±1.28 *^c^*	67.90±0.52 *^c^*	77.63±0.59 *^c^*
Cu^2+^	30.77±3.81 *^c^*	26.87±1.54 *^c^*	22.22±0.51 *^c^*	18.04±0.81 *^c^*
Fe^2+^	69.47±1.67 *^c^*	130.53±4.28 *^c^*	93.85±6.32 *^c^*	104.27±0.25 *^c^*
Fe^3+^	110.64±6.56 *^c^*	95.46±1.33 *^c^*	88.98±3.72 *^c^*	65.03±0.85 *^c^*
K^+^	105.15±3.66 *^c^*	157.85±3.84 *^c^*	108.54±3.81 *^c^*	123.10±1.36 *^c^*
Li^+^	110.28±1.04 *^c^*	94.16±2.67 *^c^*	85.53±1.19 *^c^*	97.59±2.82 *^c^*
Mg^2+^	103.77±3.10 *^c^*	104.80±4.60 *^c^*	94.05±1.91 *^c^*	107.51±3.69 *^c^*
Mn^2+^	107.05±3.98 *^c^*	93.91±2.35 *^c^*	79.74±2.39 *^c^*	75.98±1.90 *^c^*
Na^+^	108.20±0.23 *^c^*	128.07±0.22 *^c^*	103.52±1.77 *^c^*	74.84±0.78 *^c^*
Ni^2+^	113.15±6.11 *^c^*	98.25±1.10 *^c^*	95.68±3.67 *^c^*	70.68±1.57 *^c^*
Zn^2+^	86.39±5.46 *^c^*	141.25±3.12 *^c^*	87.24±3.89 *^c^*	75.57±3.31 *^c^*

a The counter of all metals was chloride. *^b^*Without metal ion added (as 100%). *^c^*Values in the same column differ significantly from values without metal added (*p*<0.05).

### *In vitro* digestibility test

**Figure 4:**
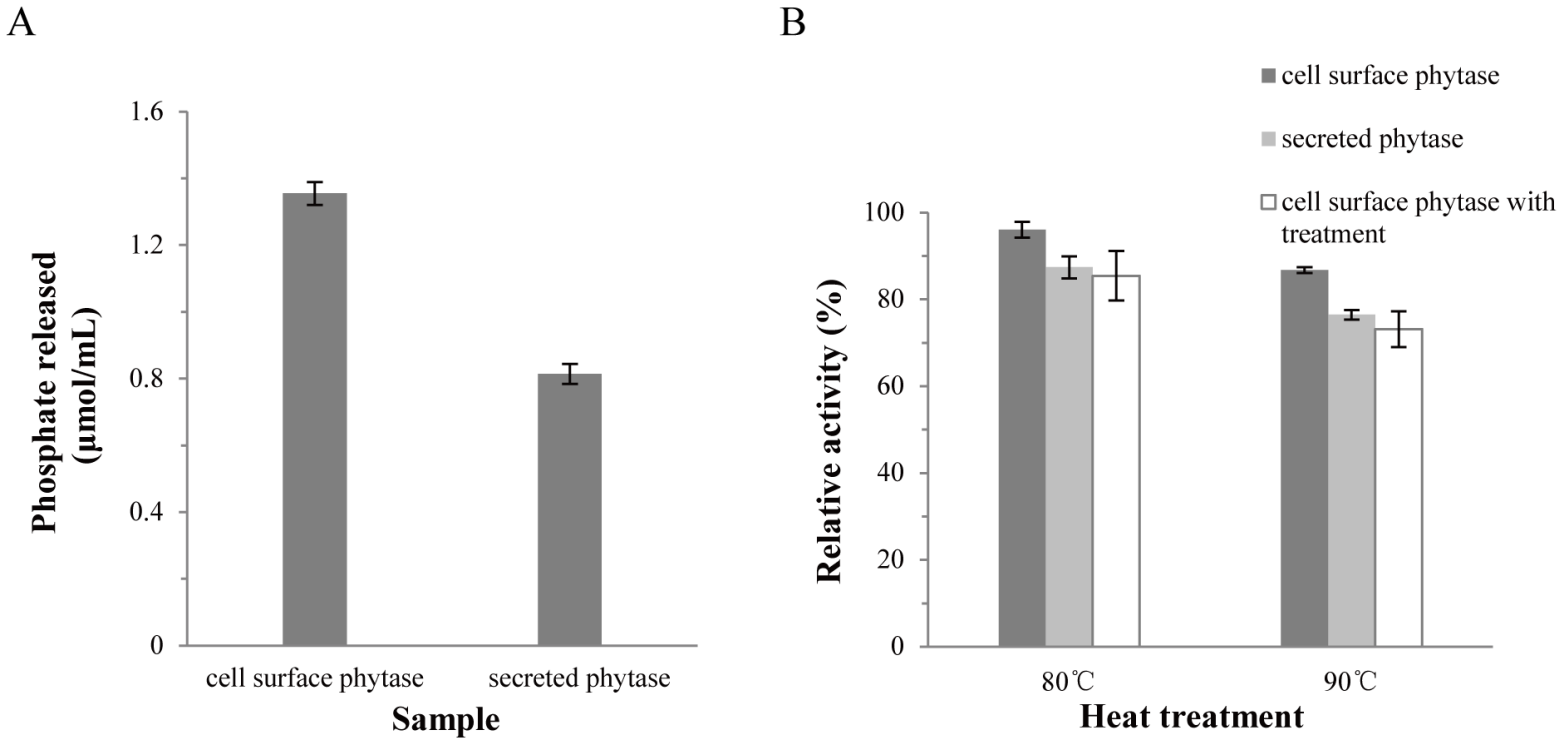
*In vitro* digestibility test of cell surface and secreted phytases. A: Measured phosphate (Pi) released from a corn-based diet mixed with cell surface or secreted phytases. B: Simulation of the pelleting process (i.e., incubation for 3 min at 80°C or 5 min at 90°C prior to activity measurement), followed by determination of the amount of released Pi. The levels of released Pi were compared with samples without heat treatment. GS115/ZαA served as a background measurement.

The amount of phosphate released from feed after incubation with cell surface phytase or secreted phytase was analyzed (Fig. 4). Using either cells or secreted phytase at 4 U of phytase activity g^−1^ feed, approximately 23% more phosphate was released by cell surface phytase than by secreted phytase (Fig. 4A). These data suggest that the cell surface phytase may be a more efficient enzyme for phytic acid digestion than the secreted version of this enzyme under conditions similar to those of the digestive tract of chickens. Moreover, when the samples were mixed with feed before heat treatment, simulating the process of pelleting (3 min at 80°C or 5 min at 90°C), a similar amount of phosphate was detected (Fig. 4B). Both the secreted and cell surface phytase had more than 70% of the activity remained simulating the process of pelleting (Fig. 4B). Interestingly, in both heat treatment experiments, the amount of phosphate released by the cell surface phytase was higher than that released by the secreted phytase. In summary, our data demonstrate that phytases exposed on the surface of yeast cells may resist high temperatures better than the secreted enzymes when mixed with feed. Furthermore, the yeast expression system is capable of folding and glycosylating heterologous eukaryotic proteins [43], [44]. In comparison with other yeasts, *P. pastoris* has the advantage of high density cultivation in inexpensive medium [36]. Therefore, the cell surface phytase of *P. pastoris* has many potential benefits and is an important potential feed supplement.

## Conclusion

Using the *P. pastoris* GPI-anchored glycoprotein homologue Gcw61p and phytase from *C. amalonaticus* CGMCC 1696, we generated a cell wall anchoring systems for protein display. The obtained data suggest that anchoring phytase with Gcw61p improves the stability of phytase in varying pH conditions and increases the efficiency of the enzyme for phytic acid digestion.

## Supporting Information

S1 Figure **Restriction enzyme digestion of plasmid Phy.** The plasmid Phy was digested using *EcoR* I and *Kpn* I.
(TIF)

S2 Figure **Restriction enzyme digestion of plasmid Phy-GCW61.** The plasmid Phy-GCW61 was digested using *Kpn* I and *Not* I. A: The results of the restriction enzyme digestion were visualized using a 1% (wet wt/vol) agarose gel. B: The results of the restriction enzyme digestion were visualized using a 2% (wet wt/vol) agarose gel.
(TIF)

S3 Figure **Colony PCR verification of GS115/Phy and GS115/Phy-GCW61.** A: Colony PCR verification of GS115/Phy. B: Colony PCR verification of GS115/Phy-GCW61.
(TIF)

S1 Table **Primers used to amplify fragments for expression cassette construction.**
(DOCX)
