# Supplementary figures and images for: Citrobacter amalonaticus Phytase on the Cell Surface of Pichia pastoris Exhibits High pH Stability as a Promising Potential Feed Supplement
Source: PLoS One. 2014 Dec 9;9(12):e114728. doi: 10.1371/journal.pone.0114728 (PMC4260871; doi:10.1371/journal.pone.0114728)

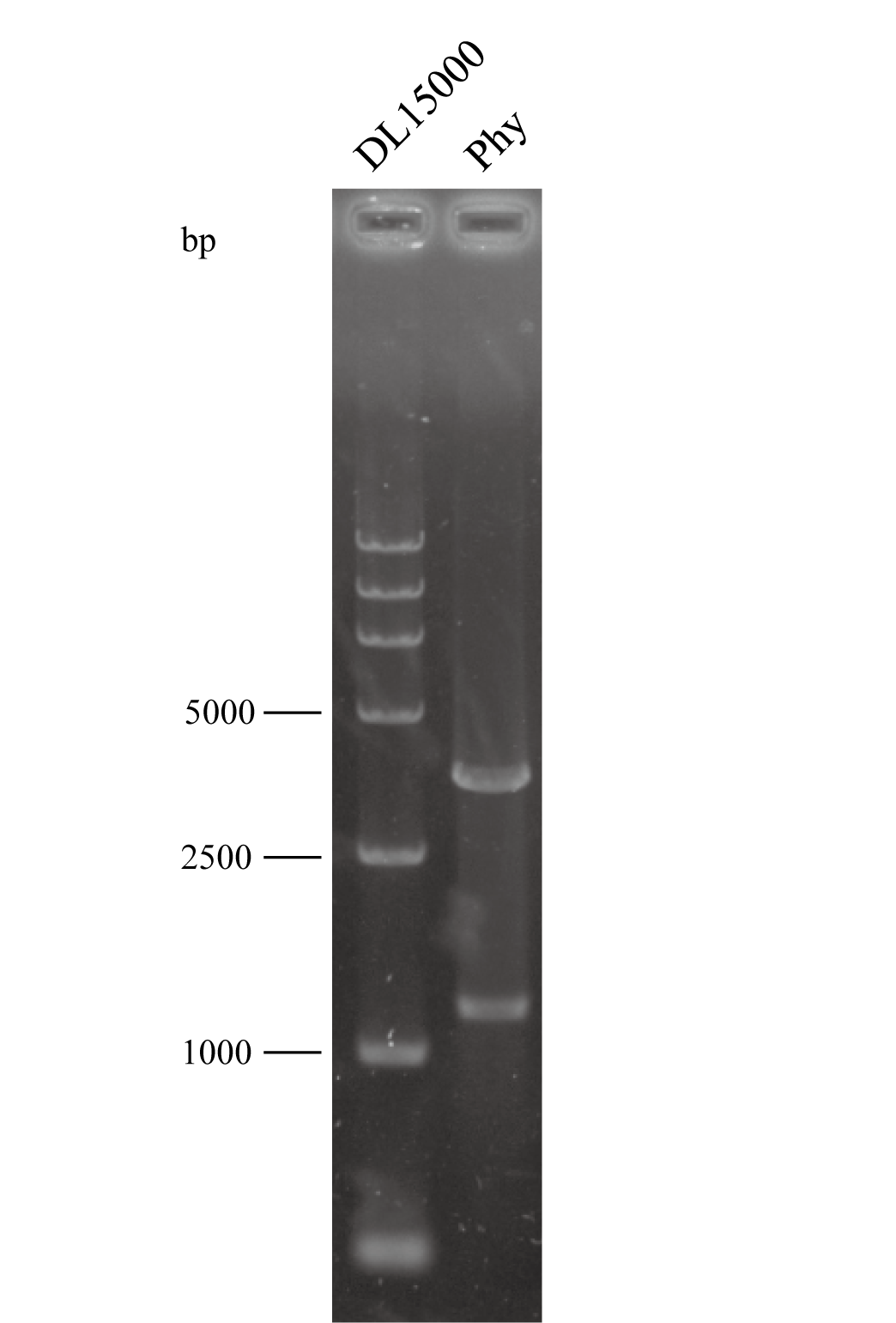

Supplement: S1 Figure — Restriction enzyme digestion of plasmid Phy. The plasmid Phy was digested using EcoR I and Kpn I. (TIF) [file pone.0114728.s001.tif]

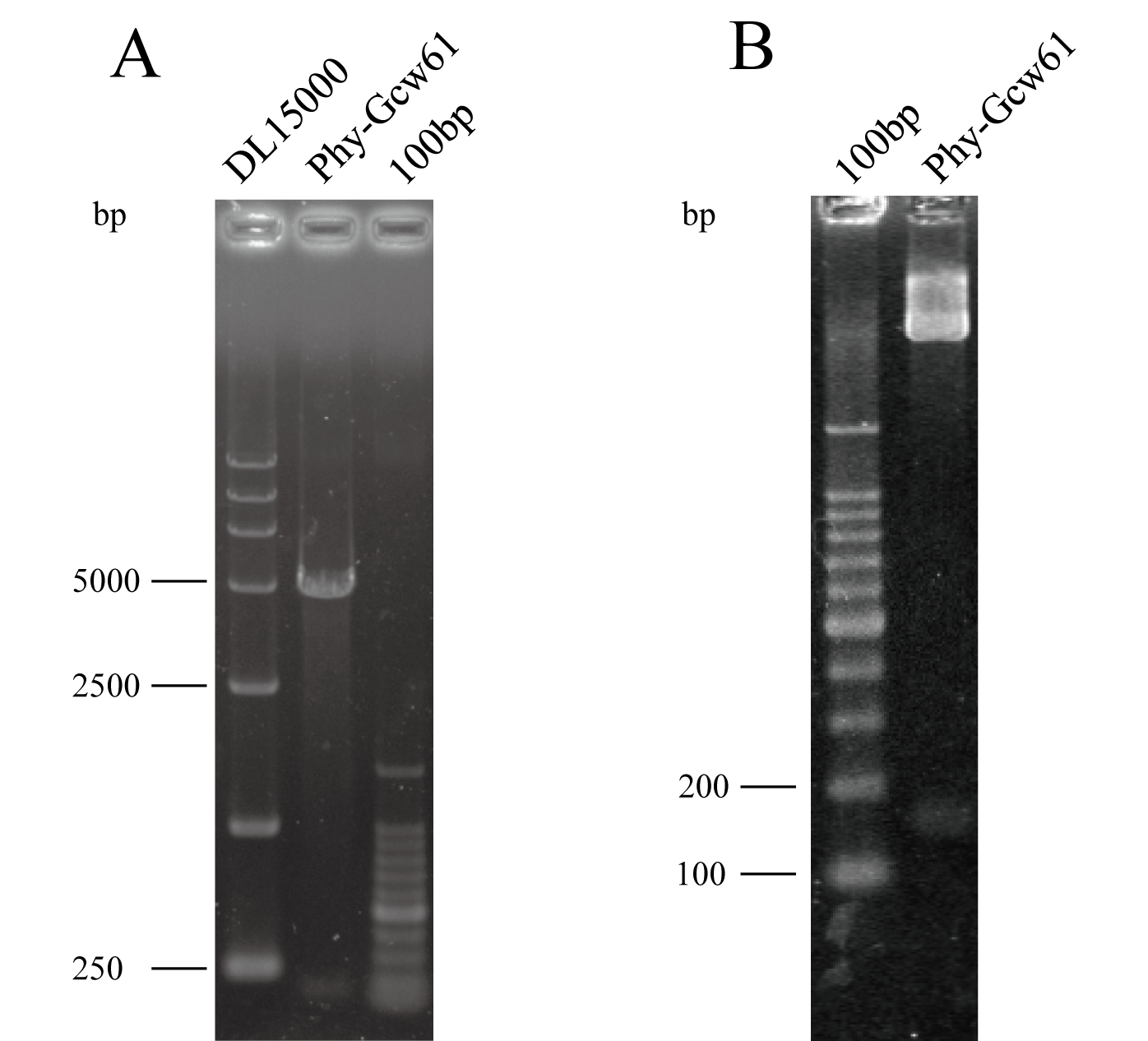

Supplement: S2 Figure — Restriction enzyme digestion of plasmid Phy-GCW61. The plasmid Phy-GCW61 was digested using Kpn I and Not I. A: The results of the restriction enzyme digestion were visualized using a 1% (wet wt/vol) agarose gel. B: The results of the restriction enzyme digestion were visualized using a 2% (wet wt/vol) agarose gel. (TIF) [file pone.0114728.s002.tif]

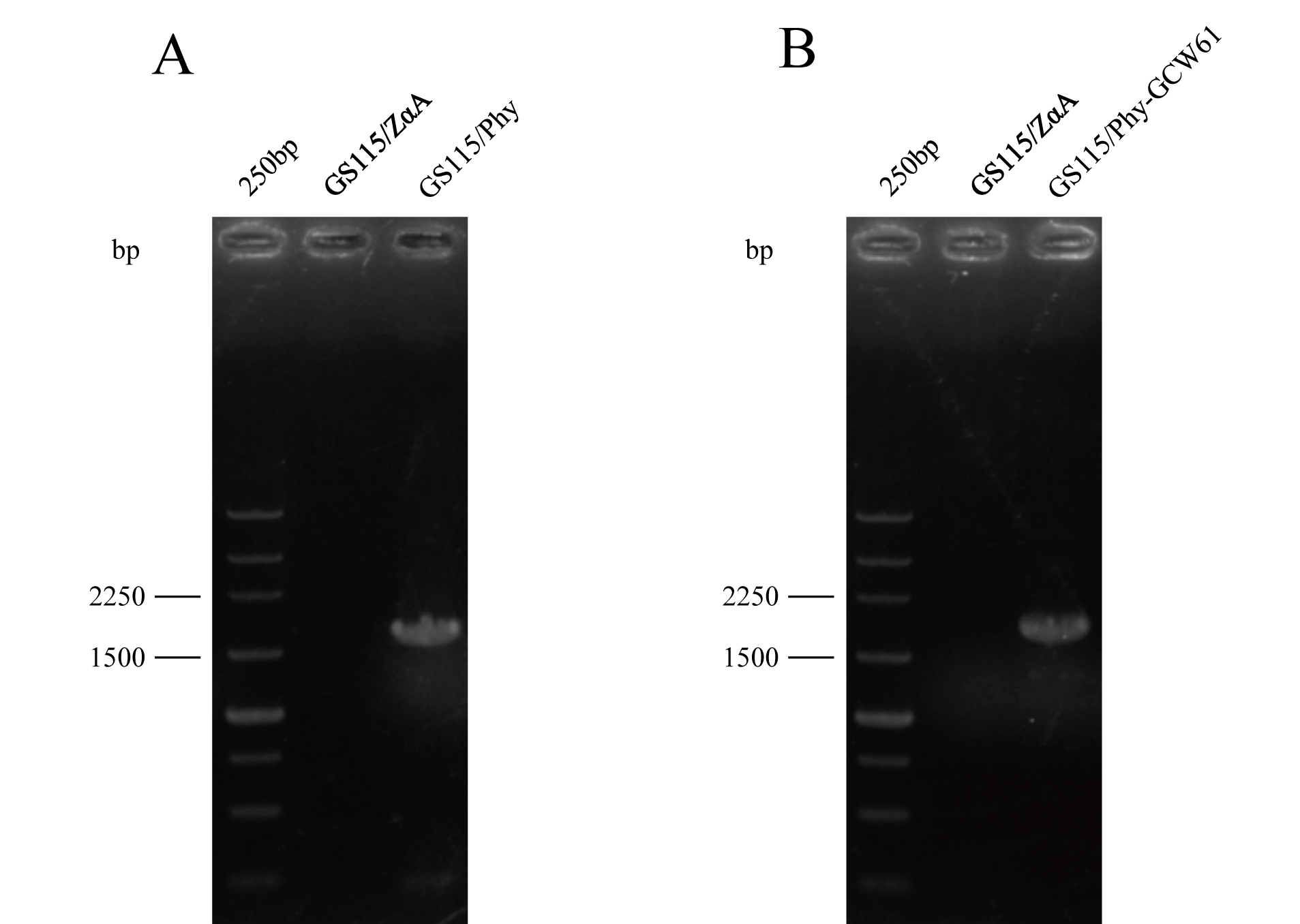

Supplement: S3 Figure — Colony PCR verification of GS115/Phy and GS115/Phy-GCW61. A: Colony PCR verification of GS115/Phy. B: Colony PCR verification of GS115/Phy-GCW61. (TIF) [file pone.0114728.s003.tif]
