# Supplementary material for: Citrobacter amalonaticus Phytase on the Cell Surface of Pichia pastoris Exhibits High pH Stability as a Promising Potential Feed Supplement
Source: PLoS One. 2014 Dec 9;9(12):e114728. doi: 10.1371/journal.pone.0114728 (PMC4260871; doi:10.1371/journal.pone.0114728)
Supplement: S1 Table — Primers used to amplify fragments for expression cassette construction. (DOCX) [file pone.0114728.s004.docx]

**Table S1.** Primers used to amplify fragments for expression cassette construction

| Primer Name | Primer sequence |
| --- | --- |
| PhyF | GCCTGAATTCGATTACAAGGATGATGACGATAAGGAGGTTCCAGACGATATGAAATTG |
| PhyR | TACGGTACCTCTATTAACGTCGGCCA |
| GCW61F | CACACGGTACCAACAACCTATCAAACGAGAGTA |
| GCW61R | TATAGCGGCCGCTTAAATCAATAGAGCAACAC |
